# Probing Multicellular Tissue Fusion of Cocultured Spheroids—A 3D‐Bioassembly Model

**DOI:** 10.1002/advs.202103320

**Published:** 2021-10-10

**Authors:** Gabriella C. J. Lindberg, Xiaolin Cui, Mitchell Durham, Laura Veenendaal, Benjamin S. Schon, Gary J. Hooper, Khoon S. Lim, Tim B. F. Woodfield

**Affiliations:** ^1^ Christchurch Regenerative Medicine and Tissue Engineering (CReaTE) Group Department of Orthopaedic Surgery University of Otago Christchurch 2 Riccarton Avenue Christchurch 8011 New Zealand

**Keywords:** 3D‐bioassembly, cartilage tissues, cocultured spheroids, high throughput, microtissues, spheroid fusion, tissue fusion

## Abstract

While decades of research have enriched the knowledge of how to grow cells into mature tissues, little is yet known about the next phase: fusing of these engineered tissues into larger functional structures. The specific effect of multicellular interfaces on tissue fusion remains largely unexplored. Here, a facile 3D‐bioassembly platform is introduced to primarily study fusion of cartilage–cartilage interfaces using spheroids formed from human mesenchymal stromal cells (hMSCs) and articular chondrocytes (hACs). 3D‐bioassembly of two adjacent hMSCs spheroids displays coordinated migration and noteworthy matrix deposition while the interface between two hAC tissues lacks both cells and type‐II collagen. Cocultures contribute to increased phenotypic stability in the fusion region while close initial contact between hMSCs and hACs (mixed) yields superior hyaline differentiation over more distant, indirect cocultures. This reduced ability of potent hMSCs to fuse with mature hAC tissue further underlines the major clinical challenge that is integration. Together, this data offer the first proof of an in vitro 3D‐model to reliably study lateral fusion mechanisms between multicellular spheroids and mature cartilage tissues. Ultimately, this high‐throughput 3D‐bioassembly model provides a bridge between understanding cellular differentiation and tissue fusion and offers the potential to probe fundamental biological mechanisms that underpin organogenesis.

## Graphical abstract

This facile bioassembly platform to study cartilage–cartilage interfaces offers a powerful route to probe multicellular fusion mechanisms in developmental stages of tissue growth and regenerative repair strategies. This hybrid 3D‐bioassembly model provides a bridge between understanding, tissue fusion and cellular differentiation by recapitulating detailed biological mechanisms in vitro—systematically unlocking the potential of tissue growth and subsequent organ engineering.

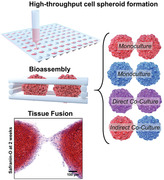

## 1 Introduction

Osteoarthritis is a prevalent and intensifying joint disorder, causing significant amount of pain, disability as well as socio‐economic costs worldwide.^[^ ^1^, ^2^, ^3^ ^]^ Using novel tissue engineering and regenerative medicine (TERM) approaches, it was initially believed that implantation of MSCs, expanded autologous HACs, or cocultures thereof, would lead to better cartilage tissue repair outcomes^[^ ^4^, ^5^, ^6^ ^]^ compared to existing surgical techniques such as microfracture or autografting. Although holding great promise, these cell‐based surgical options are not consistently successful.^[^ ^7^, ^8^, ^9^, ^10^, ^11^, ^12^, ^13^, ^14^ ^]^ They display limited long‐term efficacy and still lack clear superiority over traditional, and cheaper, surgical microfracture techniques used currently in clinical settings.^[^ ^11^, ^12^, ^15^, ^16^, ^17^, ^18^ ^]^ Two reasons for this may be that residing host chondrocytes are known to have a low metabolic turnover^[^ ^19^ ^]^ while the seeded cells often have a limited ability to stimulate functional tissue repair beyond the implant itself. Taken together, this restricts regeneration within the surrounding host tissues as well as the fusion region that forms between the implant and native tissues. Fusion is a particularly important cellular mechanism as, concomitant with temporal extracellular matrix (ECM) formation to generate functional tissue, it is one of the key phases during embryonic development and subsequently the development of anisotropic tissues and fullorgans.^[^ ^20^ ^]^

**Figure 1:**
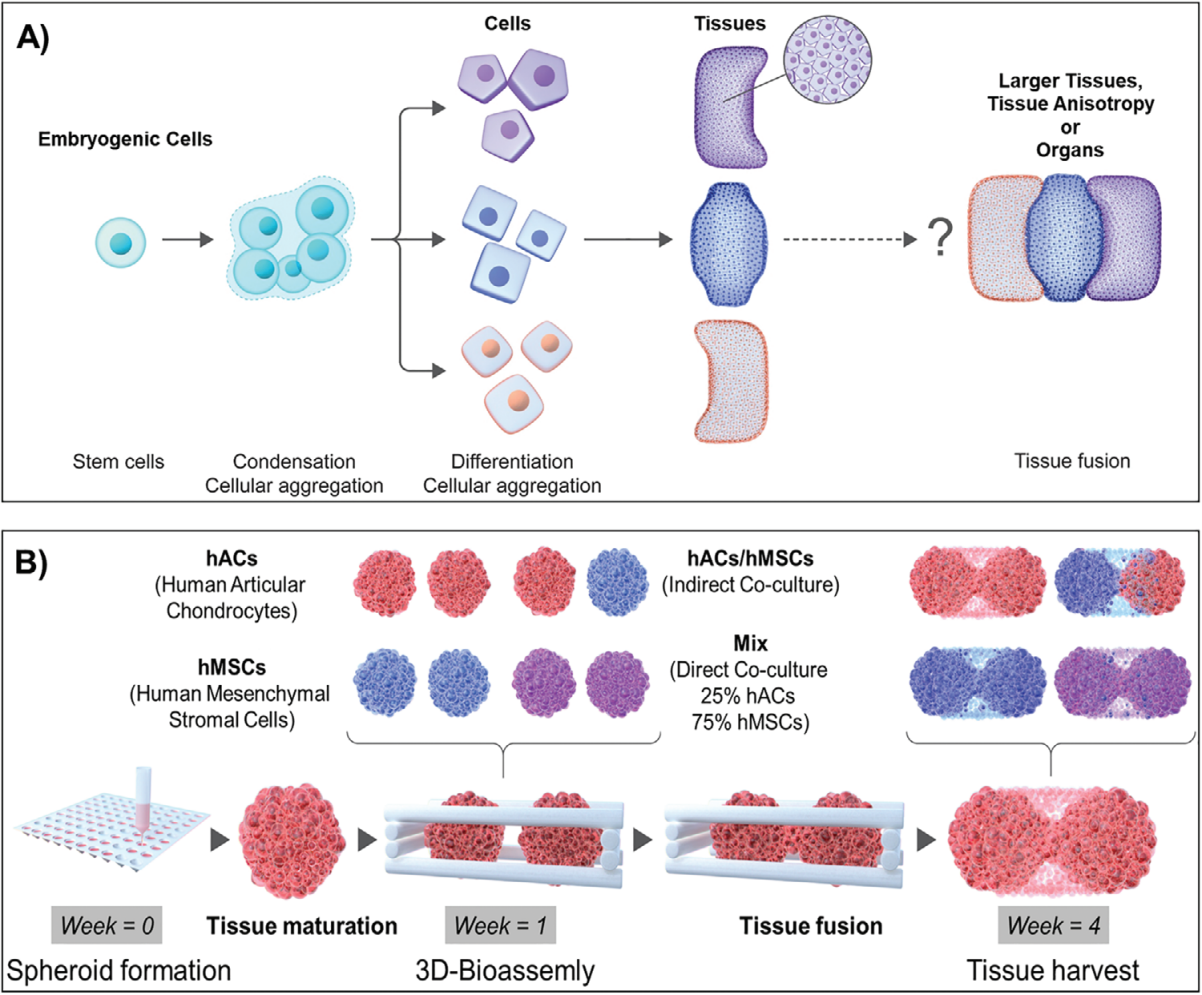
A) Illustration of a stem cell dividing and undergoing condensation prior to tissue specific differentiation and aggregation into tissue structures, and subsequent fusion into larger, matured configurations. B) The tissue fusion step is key to tackling the biological complexity of engineering larger structures, and subsequent tissue anisotropy and organogenesis. It requires reliable 3D‐models proficient to systematically screen these fusion events with precise control over microenvironmental factors and in high‐throughput. Schematic of the developed 3D‐model used for probing multicellular tissue fusion, a steppingstone towards organ‐like growth as well as cartilage integration. An established, high‐throughput centrifugation method is used to fabricate hundreds of cartilage tissue spheroids.^[^ ^25^ ^]^ After one week of preculture, two mature tissue spheroids are placed adjacently within a custom‐made, 3D‐printed thermoplastic cage. The two tissue units can be made from single‐ or multicellular sources and combined in any desired patterns, enabling the study of monocultures as well as both indirect and direct coculture mechanisms. The flexibility of this modular 3D‐bioassembly approach enables the fusion of mature hAC and hMSC spheroids into larger tissues following 3 weeks of culture, coordinated through interactions between distinct cells sources and neighboring tissue interfaces.

Little is yet known about this next phase: fusing of tissue engineered cartilage into larger structures, and ultimately fusion of additional musculoskeletal tissues to form the basis of the joint organ (**Figure** **1**A). The general principle for tissue fusion proposes that residing cells need to cleave their current tissue linkage to subsequently migrate and form new connections with the opposing tissue, also referred to as “melting together.”^[^ ^21^ ^]^ Fusion of cartilage tissue is, thus, an exceptionally difficult challenge as hyaline articular cartilage is made up of a very dense and avascular network of intertwined collagen type II and glycosaminoglycans (GAGs).^[^ ^9^, ^21^, ^22^, ^23^, ^24^ ^]^ The inherently low metabolic activity combined with the impaired ability of naturally residing chondrocytes to migrate into surrounding matrices may further explain the lack of effective tissue integration, and subsequently cell‐based regenerative repair options.^[^ ^7^ ^]^ Successful cartilage TERM approaches rely on the ability of engrafted cells to migrate between interfaces and secrete healthy signals as well as tissue rich in GAGs and collagen type II, also within the fusion region and adjacent tissues to prime functional host tissue integration.

MSCs are known to have exceptional migratory properties and have been highlighted for their capacity to also facilitate tissue regeneration by instructing adjacent articular chondrocytes (ACs) to differentiate.^[^ ^20^, ^26^, ^27^, ^28^, ^29^, ^30^, ^31^, ^32^, ^33^, ^34^, ^35^, ^36^, ^37^, ^38^, ^39^, ^40^ ^]^ A wide range of direct MSC/AC coculture ratios have been investigated over the years, often demonstrating an improved chondrogenic capacity in cocultures consisting if higher proportions of MSC (50–90%).^[^ ^41^, ^42^ ^]^ It is furthermore noted that clinical trials performed with cocultures also advocates for higher ratios of MSCs (80–90%) as it is often challenging to harvest sufficient numbers of autologous ACs to be able to perform single surgery approaches.^[^ ^26^, ^43^, ^44^ ^]^ While several studies prove that trophic factors are an important feature of MSCs, reinforcing that cocultures of MSCs and ACs are powerful platforms to induce chondrogenesis,^[^ ^20^, ^28^, ^29^, ^30^, ^31^, ^32^, ^33^, ^34^, ^35^, ^36^, ^37^, ^38^, ^39^, ^40^ ^]^ the physical extent of MSCs signaling ability remains highly controversial. Some reports advise that close proximity and direct cell–cell contact in cocultures is a prerequisite for this intracellular communication with adjacent cells to take place effectively,^[^ ^33^ ^]^ suggesting that ACs residing in surrounding tissues would not benefit greatly from MSC‐secreted signals. Others underline that indirect cocultures of MSCs and ACs, where the two cell types are engrafted separately but cultured within the same system, can still benefit from paracrine MSC signaling,^[^ ^28^, ^29^, ^30^, ^32^, ^33^, ^34^, ^35^ ^]^ instead suggesting that it is possible to extend the effect of MSC signaling also to adjacent tissues. Many of these inconsistencies found between direct and indirect coculture studies may be due to the complexity and diversity of direct cell–cell and cell–ECM interactions, as well as indirect interactions though the release of soluble factors that can occur in coculture systems, and possible combinations thereof. One factor is that many in vitro coculture models vary greatly in their capacity to recapitulate the different stages of multicellular tissue regeneration.

Another general limitation of current 3D models includes overlooking how direct and indirect cellular interactions may affect the dynamic process of tissue fusion or integration between two or more tissue components. Together, this underlines that the field needs multidirectional and scalable 3D in vitro models to study the ability of MSCs, ACs, and indirect and direct cocultures thereof, to facilitate fusion of adjacent tissues in a biological relevant environment. Understanding fusion relationships from a direct and indirect multicellular coculture is not only important from a developmental biology perspective and subsequent bioengineering of full‐scale organs,^[^ ^41^, ^45^ ^]^ it would also aid in the understanding of integrating engineered tissue constructs with surrounding host tissue following implantation in vivo.^[^ ^8^, ^9^ ^]^

In this project, we aim to design a high‐throughput 3D‐model, able to recapitulate the different stages of multicellular tissue regeneration with high biological relevance, to study the chondroinductive role of MSCs on tissue fusion mechanisms and ECM formation at cartilage‐to‐cartilage interfaces. We herein propose to adopt hybrid biofabrication platforms utilizing previously described customizable, bottom‐up, bioassembly technologies^[^ ^46^ ^]^ to design a structural thermoplastic 3D‐scaffold framework that can be populated with two distinct tissue spheroids^[^ ^25^ ^]^ to model multicellular tissue growth (Figure 1B). This specifically involved the study of underlying biological mechanisms, such as multicellular crosstalk, cell proliferation, paracrine signaling, migration, and new ECM deposition. Deconstructing these complex cartilage processes, that span several time and length scales of biological organization, provides a step‐wise reconstruction of multicellular tissue growth and tissue fusion, forming larger tissues and the basis for tissue anisotropy and downstream joint organogenesis. By applying MSCs and HACs as starting‐point to study multicellular tissue growth, we describe an in vitro 3D platform proficient to explore correlations between basic biological functions, coordination of neighboring tissue interfaces and the cellular capacity to secrete healthy hyaline cartilage tissue in the fusion regions, a capability which is imperative to successful tissue host cartilage fusion and integration, and subsequent translation of any cartilage repair strategy. Additional examples of MSC and human umbilical vein endothelial cell (hUVEC) cocultures and hydrogel support platforms described herein provides a proof‐of‐concept that the 3D‐model can readily be extended beyond cartilage to include additional joint tissues.

## 2 Experimental Section

### 2.1 Material and Reagents

Primary antibodyfor Connexion43 (C6219), ethylenediaminetetraacetic acid disodium salt dihydrate (Di‐sodium‐EDTA), sodium hydroxide (NaOH), Trizma base, 1,9‐dimethyl‐methylene blue zinc chloride double salt (DMMB), Proteinase K, 4% neutral buffered formalin, sodium chloride (NaCl), chondroitin‐4‐sulfate, glycine, phosphate buffered saline (PBS), ITS+1, l‐proline, dexamethasone, l‐ascorbic acid‐2‐phosphate sesquimagnesium salt (AsAp), hyaluronidase, safranin‐O, fast green FCF, hydrochloric acid (37%), sodium bicarbonate, and ß‐mercapto‐ethanol were purchased from Sigma‐Aldrich, St Louis, MO. CyQuant cell proliferation assay kit, Trypsin‐EDTA solution, penicillin–streptomycin (10 000 U mL^−1^ and 10 000 µg mL^−1^), bovine serum albumin (BSA), Gibco 4‐(2‐hydroxyethyl)‐1‐piperazineethanesulfonic acid (HEPES), Gibco DMEM hi‐glucose glutamax media, Gibco *α*MEM nucleosides glutaMAX, Gibco nonessential amino acids (NEAA), fibroblast growth factor 2 (FGF2), molecular probes calcein‐AM and propidium iodide, 4,6‐diamidino‐2‐phenylindole (D1306, DAPI), goat‐anti‐mouse secondary antibody (Alexa Fluor 488), molecular probes QTracker 585 and 655, QTracker 525 and 625, and Gibco fetal bovine serum (FBS) was obtained from Thermo Fisher Scientific, Auckland, NZ. Primary antibodies for collagen type II were purchased from DSHB (II‐II6B3‐C), Iowa City, USA. Primary antibodies for aggrecan (ab3773), collagen type X (Ab49945), and collagen type I (Ab34710) were purchased from Abcam, Melbourne, Australia. Transforming growth factor *β*‐1 (TGF*β*‐1) was obtained from R&D systems, Minneapolis, USA. Type II collagenase was obtained from Worthington biochemical corporation, Lakewood, USA. Acetic acid (glacial, 100%), Di‐sodium hydrogen phosphate (NA2HPO4), and Gill's hematoxylin were obtained from Merck Millipore, North Shore City, NZ. Tissue‐Tek O.C.T Compound Sakura Fintek was purchased from VWR International, Radnor, USA. PCL was purchased from PolyVation, The Netherlands.

### 2.2 Scaffold Fabrication

**Figure 2:**
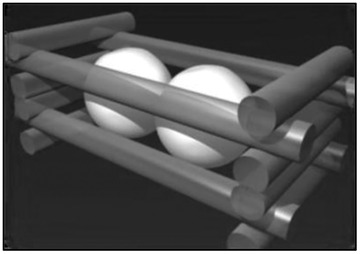
Illustration of 3D‐printed fusion cages holding two spheroids in place.

Using a Bioscaffolder (Sys+Eng), PCL polymer was heated to 120 °C, and extruded in a bilayered fashion under pressure using nitrogen gas and a screw extruder, through a 23‐guage nozzle. Polymer fibers were deposited in dual‐layer repeating 90° lattice, with fibers extruded at 0°, 90°, 0°, 0°, 90°, 90°, 0°, 0°, 90°, 0°. Fiber spacing was measured at 0.95 mm apart in the Z‐direction and 2.55 mm in the Y‐direction, and fiber diameters were 0.258 mm. The fiber spacing can be adjusted to suit specific needs of the bioassembly model, tailored to various sizes of spheroids to achieve repeatability, reliable colocation of spheroids and to prevent spheroids from moving or falling out. In this study, the length of each pore within the scaffolds was 2.292 mm, suitable for spheroids of ≈1.15 mm in diameter. Fiber spacing was 0.95 mm in the Z‐direction and 2.55 mm in the Y‐direction, and with an extruded PCL fiber diameter of 0.258 mm. The scaffolds were designed to allow for press‐fit delivery of two spheroids into the pore (**Figure** **2**), allowing for long‐term retention of the spheres within the fusion boxes, as previously detailed.^[^ ^46^ ^]^ This method resulted in 100% reliable retention of spheroids throughout all culture experiments and time points in this study. Fabricated 3D scaffolds were sterilized for at least 2 h in 70% EtOH. Scaffolds were then washed five times with PBS prior to use.

### 2.3 Cell Isolation and Expansion

Following informed consent (New Zealand Health and Disability Ethics Committees, URA/08/08/049/AM06), human bone marrow was aspirated from the center of the iliac crest. Standard Ficoll‐Paque centrifugation separation techniques was used to isolate the mononuclear fraction, as previously described.^[^ ^47^ ^]^ In short, PBS was used to diluted the bone marrow aspirates (1–4×). Using a cell strainer (100 µm), 35 mL of filtered bone marrow solution was added to 15 mL of Ficoll‐paque with care. The combined solution was centrifuged for 30 min without breaks (400 × *g*, 20 ˚C). Aspiration of the mononuclear cell layer was followed by a PBS wash and 15 min centrifugation (400 × *g*, 20 ˚C). The pelleted cells were resuspended in hMSC expansion medium (*α*MEM supplemented with 0.1 mg mL^−1^ streptomycin, 100 U mL^−1^ penicillin, 1 ng mL^−1^ FGF‐b, and 10% (v/v) FBS) and counted. 300 000 mononuclear cells cm^−2^ were plated and grown to 80% confluence (37 ˚C, 5% CO_2_). Adherent cells were further expanded to passage 3 and their multilineage potential was evaluated using three distinct adipogenic, osteogenic, and chondrogenic differentiation protocols, as previously described.^[^ ^47^, ^48^ ^]^ Differentiated cells were fixed in 4% formaldehyde and their differentiation capacity evaluated using histological protocols for chondrogenic, osteogenic, adipogenic cultures (Safranin O, Alizarin Red, and Oil Red O).

Following informed consent (New Zealand Health and Disability Ethics Committees, URB/07/04/014/AM02), healthy articular cartilage tissue was obtained from standard orthopedic surgeries (anterior cruciate ligament reconstruction). The collected tissue was digested in chondrogenic expansion medium (DMEM with 0.1 mg mL^−1^ streptomycin, 100 U mL^−1^ penicillin, 10 × 10^−3^ m HEPES, 0.4 × 10^−3^ m l‐proline, 0.1 × 10^−3^ m NEAA, 10% (v/v) FBS and 0.1 × 10^−3^ m AsAp) for 16–20 h (37 ˚C, 5% CO_2_). Using a cell strainer (100 µm), chondrocytes were separated from undigested tissue. 3000 chondrocytes cm^−2^ were plated and grown to grown to 80% confluence for three passages (37 ˚C, 5% CO_2_).

### 2.4 High Throughput Spheroid Fabrication and Culture

Spheroids were generated using a facile and reproducible 96‐well plate format that allows for high‐throughput fabrication, as previously published.^[^ ^25^, ^49^ ^]^ In brief, each well of a polypropylene 96‐well V‐bottom plate was loaded with 0.25 × 10^6^ cells in chondrogenic differentiation media (DMEM supplemented with 0.1 mg mL^−1^ streptomycin, 100 U mL^−1^ penicillin, 10 ng mL^−1^ TGF*β*‐1, 0.4 × 10^−3^ m l‐proline, 1.25 mg mL^−1^ BSA, 0.1 × 10^−3^ m NEAA, 0.2 × 10^−3^ m AsAp, 10 × 10^−6^ m dexamethasone, 10 × 10^−3^ m HEPES, and ITS+1 premix). The plate was centrifuged for 4 min (200 × *g*, RT) and cultured overnight (37 °C, 5% CO_2_). The media was refreshed at day 1 to detach the formed pellets from the plastic well plate. The spheres were cultured for 7 days until harvest for downstream applications using bioassembly strategies.

### 2.5 Hybrid 3D‐Bioassembly of Tissue Fusion Construct

**Figure 3:**
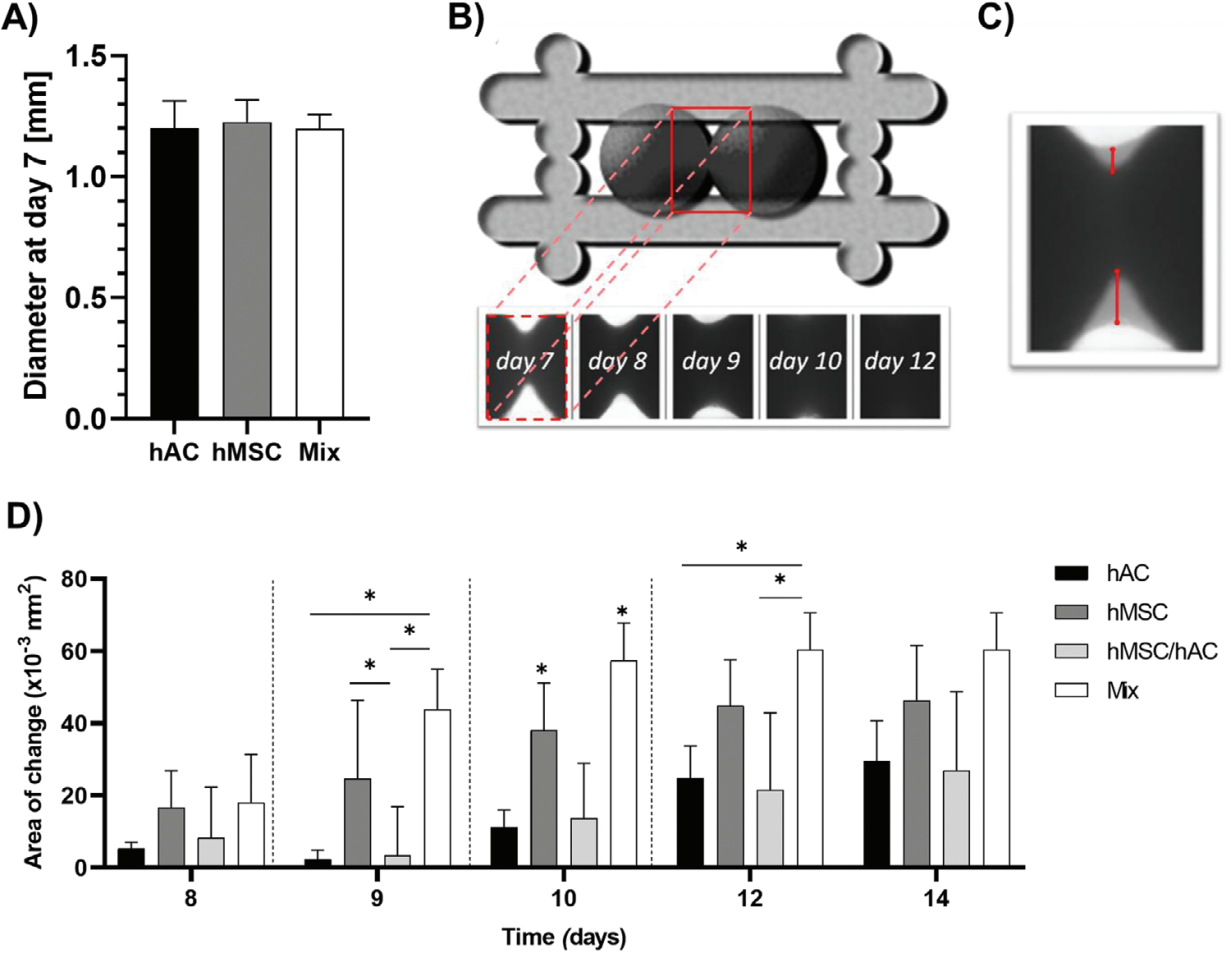
A) The size of single hAC, hMSC, and mixed spheroids were analyzed after 1 week of preculture in chondrogenic differentiation media, prior to bioassembly within the fusion boxes. B,C) Illustration of two spheroids placed adjacently within a fusion box (B, top) with zoomed in images of an experimental example of the mixed spheroid tissue–tissue interfaces as a function of time (B,C), i.e., area of change. D) Quantification of the area of change as a function time and culture conditions.

A 1 mL adjustable pipette was used to carefully handle and transfer precultured (1 week) spheroids from well‐plates to 3D plotted scaffolds, a process which has recently been automated for high‐through put screening.^[^ ^46^ ^]^ Due to inherent size variation of the spheres, ranging from ≈1.1 to 1.3 mm in diameter in this study (**Figure** **3**A), two small spheres placed adjacently would yield a 100 µm gap between the tissue modules in the ≈2.3 mm long box. Each construct was thus inspected under a brightfield microscope following assembly and the location of the spheres was adjusted with a sterile pipette when required to ensure adjacent and close contact for all samples. Four different groups were built up with two cell pellets:

1. HAC group: two 100% hAC pellets
2. HMSC group: two 100% hMSCs pellets
3. HAC/hMSC group (indirect coculture): one pellet with 100% hACs and one pellet with 100% hMSCs
4. Mix group (direct coculture): two pellets were hMSCs (75%) and hACs (25%) were mixed together.

The 3D‐bioassembled constructs were cultured for an additional 3 weeks in chondrogenic differentiation media at 37 °C, 5% CO_2_, accumulating to 4 weeks of total culture time (Figure 1B).

### 2.6 Fusion of Microtissues

Throughout the culture period, digital images of spheroids were captured with a brightfield microscope (Olympus CKX41, Japan). Individual spheroid sizes were measured using ImageJ software (version 6.1 Fiji, National Institutes of Health). Images of bioassembled samples were cropped to the region of interest and thresholded to remove background using ImageJ. The area of change (Figure S6C, Supporting Information) was measured by image subtraction of later (Figure S6B, Supporting Information) from earlier time point thresholded images (Figure S6A, Supporting Information), followed by built in ImageJ commands (“analyze particle”).

### 2.7 Cell Tracking

Cells were concentrated to 10^7^ cells mL^−1^ and incubated 90 min (37 °C, 5% CO_2_) in expansion media containing Qtracker 625 labeling solution (100 µL cell suspension mixed with 200 µL media and 2 µL labeling solution at 10 × 10^−6^ m), in accordance with manufacturer's instructions. Cells were subsequently washed twice with expansion media prior to downstream spheroid formation and bioassembly. Bioassembled constructs were collected after 1, 2, and 4 weeks of accumulated culture. The samples were washed in PBS prior to fixation in 4% formaldehyde (1 h, RT). 0.3 m glycine dissolved in PBS was subsequently used to minimize free aldehyde groups following fixation. Samples were allowed to soak in OCT overnight (4 °C) prior to freezing and cryosectioning (30 µm). Fluorescently tagged cells were visualized with a Zeiss Axioimager Z1 microscope.

### 2.8 Biochemical Analysis

Following chondrogenic differentiation, bioassembled scaffolds were collected after 1, 3, and 4 weeks for analysis. The samples were solubilized using proteinase K digestions (56 °C, 1 mg mL^−1^) in a 1 × 10^−3^ m EDTA/10 × 10^−3^ m Tris‐HCl buffered solution. For GAG analysis, the samples were diluted to be within the linear range of the CS‐A standard curve and allowed to react with a pH 3 DMMB solution, as previously described.^[^ ^25^ ^]^ For DNA analysis, the samples were pretreated with DNase‐free RNase A (135 Kunitz mL^−1^) for 1 h at RT prior to analysis using the CyQUANT Kit in accordance with the manufacturer's instructions, as previously described.^[^ ^50^ ^]^

### 2.9 Histology and Immunofluorescence Examination

Cryosectioned samples were stained with Safranin‐O, fast green, and hematoxylin to visualize secreted GAGs, collagens, and the location of cellular nuclei's. Specific collagen types I, II, and X, the GAG aggrecan and gap junction protein connexin 43 were furthermore visualized through immunohistochemistry as previously described.^[^ ^47^ ^]^ In brief, 0.2% hyaluronidase was used for epitope retrieval (30 min, RT). Slides were subsequently blocked using 2% BSA (30 min, RT) to minimize unspecific antibody binding. Primary antibodies were diluted in 2% BSA blocking buffer (collagen type I – 1:200, collagen type II – 1:200, collagen type X – 1:500, aggrecan – 1:50, and/or connexin 43 – 1:200) and incubated with the samples (3 h, RT). Unbound primary antibodies were washed away prior to application of secondary antibodies Alexa Fluor 488 and Alexa Fluor 594 (1:400 dilution, 1 h, RT). DAPI was applied to further visualize the cellular nuclei's (1:1000 dilution, 10 min, RT). Images were captured using a Zeiss Axioimager Z1 microscope. Fluorescent intensity in the fusion region was quantified using ImageJ software (version 6.1 Fiji, National Institutes of Health). Both the whole construct and the fusion region was selected, respectively, using the drawing tool and the integrated density was measured using the built in ImageJ functions. A region without any cells were selected and used to subtract background fluorescence. The corrected fluorescence intensity (CFI) was calculated asfollows

(1) $$\mathrm{CFI}=\frac{ID_{\text{fusion}\;\text{region}}}{A_{\text{fusion}\;\text{region}}}-\frac{ID_{\mathrm{background}}}{A_{\mathrm{background}}}$$

where ID is the integrated density and *A* is the area of the measured region of interest.

### 2.10 Gene Expression

Following 4 weeks of culture, bioassembled spheroid samples were collected and solubilized using a proteinase K solution (10 mg mL^−1^, 30 min, 55 °C). The scaffolds were removed and the digests were centrifuged for 10 min (4 °C, 12 000 × *g*). 1 mL TRIzol regent was added to the collected supernatant and RNA was isolated following manufacturer's instructions. Briefly, 200 µL chloroform was mixed vigorously with each sample followed by 15 min centrifugation (4 ˚C, 12 000 × *g*). The RNA containing supernatant was added to 500 µL isopropanol (RT, 20 min) followed by centrifugation (15 min, 4 °C, 12 000 × *g*). The resultant RNA pellet was washed in 70% ethanol (−20 °C), dried and resuspended in water. Following the manufacturer's description, Ambion DNA‐free DNase Treatment was applied prior to spectrophotometric (Thermo Scientific, NanoDrop 8000) and spectrophotometric analysis (Agilent Technologies, 2200 TapeStation). Reverse transcription of the RNA (300 ng) into complementary DNA was completed with TaqManTM first strand synthesis followed by real‐time PCR (qRT‐PCR, Roche, LightCycler480 II) using KiCqStart SYBR Green Primers (Sigma‐Aldrich.^[^ ^47^ ^]^ Expression of genes for SOX9 (NM_000346), aggrecan (NM_001135), collagen type IA1(NM_000088), and collagen type IIA1 (NM_001844) was analyzed together with housekeeping gene Glyceraldehyde‐3‐phosphate dehydrogenase (GAPDH, NM_002046). Primer efficiency and threshold cycle were analyzed and data were collected in duplicates for each sample. The mRNA expression was normalized to the GAPDH reference gene, and pure hAC spheroid samples were used as the baseline to calculate the relative gene expression.

### 2.11 Wider Utility and Future Outlook of the 3D Tissue‐Fusion Model

HUVECs were purchased from ATCC and cultured in accordance with manufacturer's instructions. Photopolymerisable GelAGE, GelNOR, and HepSH were synthesized in accordance with previously described protocols.^[^ ^50^, ^51^, ^52^ ^]^ Precursory solutions were fabricated into cell laden (15 × 10^6^ hACs mL^−1^, 5 × 10^6^ hMSCs mL^−1^ or 5 × 10^6^ hUVECs mL^−1^) spherical issue structures using previously described microfluidics methods.^[^ ^46^ ^]^ Biomaterial‐based spheres were placed within 3D‐printed thermoplastic scaffolds either adjacently to another biomaterial‐based sphere or a mature HAC spheroid and cultured in chondrogenic differentiation media (hAC laden GelAGE‐HepSH: 5 weeks, hMSC or hUVEC laden GelNOR: 1 week).

HNCs were obtained from septal cartilage, isolated via collagenase digestion, and expanded to passage two. Expanded HNCs, MSCs or coculture combinations of the two were centrifuged using a high‐throughput 96‐well plate format (0.25 × 10^6^ cells per sphere) to form tissue spheres, and cultured in chondrogenic differentiation media. Cells were fluorescently labeled with QTracker 585 and 655 or QTracker 525 and 625 to label the different cell types within and/or between pellets following manufacturer's instructions. Following 7 days of preculture, HNC and MSC spheroids were pressed into defined pores within 3D plotted PEGT/PBT polymer scaffolds, and in a number of configurations to demonstrate control of the process, and possible applications using fluorescence imaging.

### 2.12 Statistical Analyses

Unless otherwise stated, experimental groups were prepared with three replicates and three repeats (*n* = 3, *N* = 3). One‐way ANOVA with post hoc Tukey analysis (GraphPad Prism 8) was used to assess differences between experimental groups. Unequal variances *t*‐test (Welch's *t*‐test) was applied to compare between time‐points within the same experimental groups. Unless otherwise stated, the presented data were reported as the mean value ± standard deviation. When quantifying the strength of evidence, the deviation was considered significant when *p* < 0.05 for all statistical analyses.

## 3 Results

### 3.1 Spheroid Size and Fusion Kinetics

HMSCs, hACs, and a mixture thereof, were successfully fabricated into Ø1.2 mm mature cartilage tissue modules by following cellular condensation mechanisms similar to those found during the developmental stages of cartilage growth. Two distinct spheroids were successfully 3D‐bioassembled into the customized scaffolds (Figure 1B), yielding four unique tissue interfaces: 1) hACs/hACS, 2) hMSCs/hMSCs, 3) mixed/mixed (direct coculture), and 4) hMSCs/hACs (indirect coculture). The size of individual spheroids was observed to be the same across the different compositions, measured to 1.20 ± 0.11 mm for hACs, 1.23 ± 0.09 mm for MSCs, and 1.20 ± 0.06 mm for the mixed samples (Figure 3A). Brightfield images of 3D‐bioassembled constructs, and subsequent image analysis of the region of interest (Figure 3B,C), revealed that both hMSC (45 ± 11 mm^2^) and mixed (60 ± 9 mm^2^) conditions rapidly increased their fusion area while hAC (25 ± 8 mm^2^) and hMSC/hAC (21 ± 20 mm^2^) displayed a smaller region forming after 12 days of accumulated culture (Figure 3D). Interestingly, the inclusion of hMSCs with hACs in the mixed condition can significantly increase the growth of the tissue fusion front and area of change comparing to hAC alone or the indirect hMSC/hAC coculture condition where the two cells types are initially separated.

### 3.2 Cell Migration

**Figure 4:**
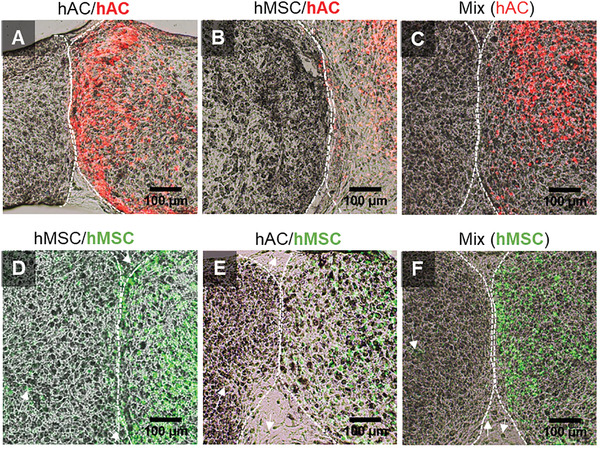
Histological sections through the center of samples fused following 4 weeks of culture in chondrogenic differentiation media. One cell type at a time has been labeled with a fluorescent tracker and is represented by two distinct colors (hAC: red; hMAC: green). The florescent images have been overlaid onto brightfield images. HACs (red) were not detected in the fusion zones of either of the A) hAC/hAC, B) hMSC/hAC or C) mixed conditions. D–F) hMSCs (green) instead displayed a high migratory capacity and were found present in most regions of the newly secreted matrix.

As demonstrated by fluorescent labeling of cells, hMSCs were rapidly able to migrate into the fusion region, and also far into to the adjacent tissue pellet (**Figure** **4**D–F; Figure S1, Supporting Information), while hACs remained mostly static (Figure 4A–C). It was furthermore observed that the tissue–tissue interface of hACs largely lacked cellular nuclei (Figure 4A), while direct (Figure 4B) and indirect (Figure 4C) cocultures denote cells in the fusion region which are not fluorescently labeled hACs—indicating stromal cell migration.

### 3.3 Matrix Formation

DNA was quantified using the CyQuant assay to indicate proliferation while the spectrophotometric assays DMMB was applied to quantify GAG excretion. The GAG production was normalized to the DNA readings to study chondrocyte differentiation capacity. The ECM secreted by encapsulated cells was visualized using standard histological‐ and immunofluorescent techniques following 4 weeks of in vitro culture in TGF‐*β* supplemented chondrogenic differentiation media.

**Figure 5:**
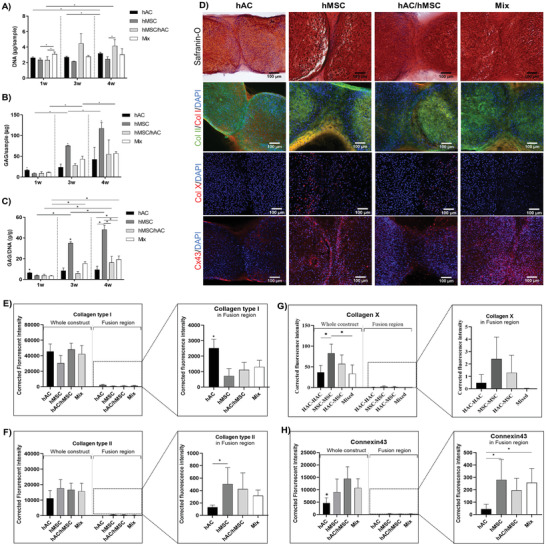
Macroscale biochemical quantification of A) DNA, B) GAG, and C) GAG/DNA content of each of the four fusion conditions as a function of time (1, 3, and 4 weeks of culture). D) Visualization of safranin‐O/hematoxylin/fast green, DAPI (blue)/collagen type II (green)/collagen type I (red), DAPI (blue)/collagen type X (red), and DAPI (blue)/connexin 43 (red) stained cryosections of spheroids after 4 weeks of accumulative culture. E–H) Quantification of florescent intensity in the overall construct and fusion region, respectively. Error bars represent the mean ± SD of six samples. * indicates significant difference (*p* < 0.05).

The biochemical analysis of the DNA content revealed that minor proliferation occurred in both pure hAC and indirect hAC/hMSC cultures, respectively (**Figure** **5**A). After 4 weeks of culture, indirect coculture of hACs/hMSCs (4.2 ± 0.8 µg) had accumulated significantly more DNA compared to pure hMSC cultures (2.4 ± 0.3 µg) while no difference was recorded between any of the other conditions. Interestingly, only at the time point of bioassembly, after 1 week preculture of individual tissue modules, a slight increase in DNA was detected in the mixed, direct coculture, condition (3.1 ± 0.1 µg) compared to the other three conditions (2.3 ± 0.4 µg).

Biochemical analysis of the GAG content further revealed the early onset of matrix formation in hAC cultures, which was greatly surpassed by hMSCs at later time points (Figure 5B). Normalizing the GAG deposition to DNA content, the chondrogenic potential was observed to be significantly upregulated as a function of time in all conditions but the hACs (Figure 5C). Interestingly, no significant difference was observed between direct (mixed) or indirect (hAC/hMSC) cocultures after 4 weeks of fusion in terms of macroscale GAG content. It was noted, however, that the mixed condition deposited significantly more ECM proteins compared to pure hAC cultures. Visual inspection of the matrix quality revealed a similar pattern, where hACs were observed to deposit more of a fibroblastic matrix rich in collagen I in the fusion region while the inclusion of hMSCs contributed to a more collagen II rich tissue (Figure 5D). This was further confirmed through quantification of the florescent intensity in the fusion region (Figure 5E–H). Looking at the construct as a whole, no difference in collagen type I or II secretion can be detected between the four conditions. Interestingly, it was noted that the mixed condition with direct‐coculture of hACs and hMSCs displayed more fibrocartilage‐like tissue formation in the fusion region early on, which was later remodeled to collagen II rich matrix after 4 weeks of accumulative culture (Figures S2 and S3, Supporting Information). In Figure 5D, it is furthermore observed that the fusion of hAC spheroids yields a hypocellular interface region, lacking the presence of cell nuclei. Again, highlighting that hAC‐based cartilage fused through tissue outgrowth while hMSC‐based cartilage fused through migration based tissue growth.

Additional histological analysis revealed that hACs deposited minimal collagen type X (Figure 5D), with no signs of hypertrophic development following 4 weeks of culture. While it was noted that MSCs secreted collagen type X within monoculture pellets, minimal to no deposition was recorded within the fusion region (Figure 5G). When indirectly cocultured with hACs, it was further observed that collagen type X was primarily deposited within the MSC‐based spheroid, and not the hAC tissue (Figure 5G). Interestingly, when MSCs are directly cocultured with hACs (mixed), barely any collagen type X disposition was detected (Figure 5D,G). These results exemplify that the benefits of direct cocultures are multidirectional, also benefitting the phenotypic stability of MSCs. Additional histological analysis demonstrated that hMSCs activates gap junction connections to a greater extent than hAC (Figure 5D,H). Subsequently, a more intense Cx43 fluorescent signal could be detected also in the mixed condition, as compared to the pure hAC culture.

### 3.4 Gene Expression

**Figure 6:**
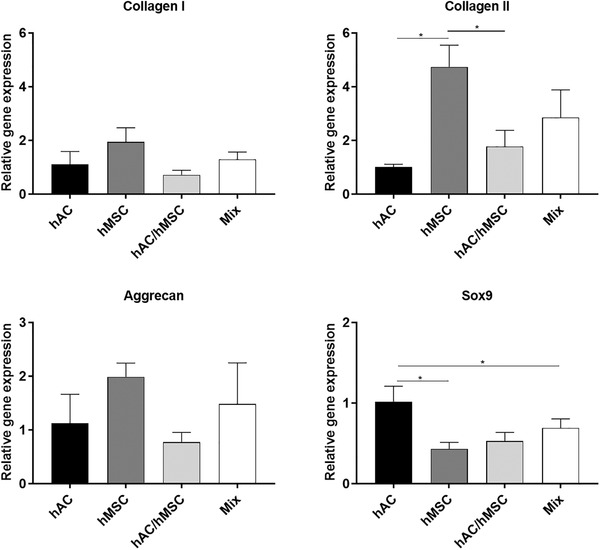
Gene expression of common markers of fibroblastic and hyaline cartilage formation in fused spheroids after 4 weeks of accumulative culture. A housekeeping gene was used to normalize values (GADHP). The quantified gene expression is presented as relative values to hAC spheroids. * indicates significant difference (*p* < 0.05).

Samples were cultured for a total of 4 weeks and analyzed using standard polymerase chain (PCR) reaction to study the presence of hyaline‐ and fibrocartilage specific genes within the whole construct. There was no significant difference in aggrecan or collagen I gene expression between any of the four culture conditions (**Figure** **6**). It was instead observed that fusion of hMSC spheroids yielded significantly higher collagen type II gene expression compared to both pure hAC and indirect cocultured hAC/hMSC spheroids, respectively. It was furthermore observed that hAC spheroids enhanced the expression of the SOX9 gene compared to pure hMSC and indirect cocultured hAC/hMSC spheroids, respectively.

### 3.5 Wider Utility and Future Outlook of the 3D Tissue‐Fusion Model

**Figure 7:**
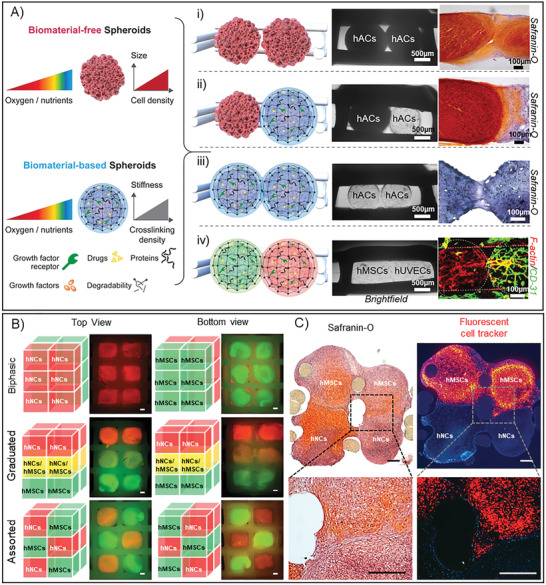
A) The developed 3D‐bioassembly model was applied with a wide range of tissue units, introducing both GelAGE (ii, iii), GelNOR (iv), and HepSH (ii, iii) as biomaterials to help gain control over the delivery of bioactive factors as well as mechanical properties during the study of tissue fusion. The proof‐of‐concept studies demonstrated that the developed 3D‐model is compatible with both biomaterial free spheroids (i) and biomaterial based spheroids (ii, iiii), which can be applied with a variety of cells: hACS (i–iii), hMSCs (iv), and hUVECs (iv). The developed 3D‐model herein sets the stage for the next tissue engineering era, advancing from tissues to organoid cultures, by providing a robust and flexible platform to study the link between cellular differentiation, tissue fusion, organ growth as well as host tissue integration. The developed 3D‐bioassembly model was further printed into a larger model, offering the ability to achieve greater throughput as well as fusion in both lateral and horizontal directions—simultaneously. B) As a proof‐of‐concept, the models were designed to hold up to 12 tissue modules. Several combinations of 7 days precultured tissue modules made from human nasal chondrocytes (HNC, red fluorescent tag) and human MSCs (green fluorescent tag) were bioassembled into biphasic, graduated, and assorted structures and cultured for 14 days (B). The constructs were imaged using fluorescent microscopy, highlighting the modular capability of the platform, scale bars = 100 µm (B). C) Two fluorescently tagged hMSC tissue modules and two nontagged hNC spheroids were furthermore precultured for 7 days followed by 14 days of bioassembly culture, scale bars = 100 µm. The constructs were imaged using brightfield and fluorescent microscopy, demonstrating tissue fusion and migration in both *X* and *Y* and directions (C).

Three distinct gelatin‐based biomaterials and three different cell types was successfully fabricated into spherical tissue modules and assembled within a fusion cage. These results display a wider applicability of the developed 3D‐model through the fusion of biomaterial‐based spheroids into larger structures from multiple cell‐ and tissue‐types, introducing additional tools to customize and mimic the native physiological microenvironments of tissues (**Figure** **7**A). HAC‐laden GelAGE and HepSH were successfully placed adjacently to a mature HAC spheroid (biomaterial‐free, Figure 7A ii) or another GelAGE‐HepSH sphere (Figure 7A iii). Following long‐term culture (5 weeks), it was observed that the GelAGE‐HepSH can fuse with adjacent biomaterial‐based spheres to make up a larger structure, offering a controllable step toward tissue growth (Figure 7A ii). It was furthermore observed that the biomaterial‐based spheres fused with mature, biomaterial‐free HAC‐spheres, mimicking the process of integration between engineered tissues and the host tissue (Figure 7A iii). In addition, the results showcased that the applicability of the platform is not limited to a specific tissue type, exemplified by the study of two distinct tissue spheroids, fabricated from GelNOR), fusing to form vascularized networks following indirect coculture of hMSCs and hUVECs (Figure 7A iv).

The 3D‐printed fusion cages were further upscaled into two‐layered constructs able to hold either 4 or 12 tissue modules within a single construct. Two distinct cell sources, human nasal chondrocytes (HNCs) and human MSCs were precultured and successfully bioassembled within these larger 3D‐models. Results showcased that the applicability of the platform is not limited to a specific number of tissue spheres or specific placement. Both direct and indirect coculture strategies were successfully used to bioassemble biphasic, graduated and assorted structures of HNCs and MSCs (Figure 7B). Overlay fluorescently images demonstrate the controlled placement of distinct nanoparticle labeled tissue modules in arrayed structures. The graduated structures, combining both direct and indirect cocultures, further highlight the ability of the platform to screening multidirectional fusion mechanisms (*X*, *Y*, and *Z* directions) in 3D within one construct—enabling a more high‐throughput workflow. Graduated constructs further provide a proof‐of‐concept that the 3D‐model can be used to also study the development of tissue anisotropy. In addition, results demonstrate that the MSCs within mature cartilage tissue modules were able to migrate and fuse in both lateral and longitudinal directions following arrayed bioassembly (Figure 7C).

## 4 Discussion

### 4.1 Cartilage Tissue Fusion—A Proof of Concept Model

To investigate fusion‐kinetics and ‐mechanism of multicellular cartilage tissue modules, we developed a facile hybrid 3D‐bioassembly model. During optimization of the 3D‐bioassembly process, it was observed that 1.0–1.2 mm spheroid cultures are a reliable approach to form mature, dense cartilage tissues in high‐throughput for downstream applications from a variety of clinically relevant cell sources. The lack of ECM secretion at the center of larger pellets has been reported as a problem in previous studies,^[^ ^45^, ^53^, ^54^ ^]^ mainly due to limited transport or diffusion of oxygen and nutrients.^[^ ^55^ ^]^ However, lower oxygen levels are known to increase chondrogenic metabolic activity given human articular cartilage is normally exposed to a hypoxic microenvironment.^[^ ^56^ ^]^ This may contribute to the stability and functional tissue formation also at the core of the relatively large (Ø1.2 mm) cartilaginous spheroids applied in this study. Li et al. specifically demonstrated that cartilaginous spheroids that are large enough to induce an oxygen drop below 8% induces GAG secretion during normoxic cultures (21% O_2_).^[^ ^57^ ^]^ This is endorsed by several other literature examples, including our own work.^[^ ^25^, ^46^, ^57^, ^58^, ^59^, ^60^, ^61^, ^62^, ^63^ ^]^

In addition to spheroid diameter, the change in tissue–tissue interface area was quantified,^[^ ^64^, ^65^ ^]^ revealing a rapid change of fusion area in the hMSC and mixed conditions following early upregulation of cell migration and tissue formation. Cellular migration is known to represent a fundamental process for both tissue homoeostasis and repair throughout our lives.^[^ ^66^ ^]^ As the results revealed that tissue outgrowth without cell migration was the main contributor for hAC tissue fusion, the addition of hMSCs, with demonstrated capability to migrate to surrounding tissues, may thus be critical for achieving more rapid and integrative cartilage repair strategies. These observations align well with the widely established paradigm that stem cells have an remarkable, inherent ability to migrate^[^ ^67^ ^]^ while chondrocytes are known to exhibit an impaired ability to both migrate and integrate with surrounding tissues as they are typically surrounded by a very dense ECM.^[^ ^68^ ^]^ Chondrocytes are instead known to communicate with each other mainly via diffusible signals, such as secretion of proteins, peptides, growth factors, and nucleosides, rather than direct cell‐to‐cell contact.^[^ ^69^ ^]^ It should be noted, however, that chondrocytes can display an increased cell migration behavior if dedifferentiating toward a fibroblastic lineage^[^ ^70^, ^71^ ^]^ and/or if surrounded by less mature cartilage matrix.^[^ ^68^ ^]^ The limited mobility of chondrocytes observed in this study, also in coculture with hMSCs, thus highlights that the in vitro model developed herein is able to replicate healthy, mature tissue–tissue migration and fusion mechanisms and can be utilized for high‐throughput screening of various TERM strategies.

Following DNA quantification, it was noted that cells in the mixed condition proliferated during the 1 week preculture period. It should be underlined that the indirect hAC/hMSC coculture condition at the 1 week time point has not yet experienced a multicellular environment. The early increase in DNA in the mixed condition is likely due to coculture‐induced proliferation driven by cell–cell contact between hACs and hMSCs or communication though soluble extracellular vesicles as no difference could be detected between pure hAC and MSC cultures.^[^ ^36^, ^38^ ^]^ The onset of proliferation in the indirect hAC/hMSC cocultures was slower and could first be observed after 4 weeks of culture. Although it is difficult to make direct comparisons across coculture studies, due to complex factors such as donor variability, it has previously been suggested that direct cocultures of hAC and hMSC can result in hMSC‐induced chondrocyte proliferation.^[^ ^72^ ^]^ Our study indicates that culturing different cell populations in distinct hierarchical zones, without initial close contact, can modulate the extent of cell proliferation over time, whereas direct and close exposure to paracrine signaling between mixed cell sources accelerates proliferation early on, simply due to increased exposure to multicellular factors. Chondrocytes transitioning to a fibroblast‐like phenotype typically display high cellular proliferation rate and low GAG production^[^ ^73^, ^74^, ^75^, ^76^ ^]^ while hMSCs are known to undergo a series of sequential biological processes to regenerate musculoskeletal tissue: condensation, overt differentiation, proliferation, maturation, hypertrophy, and finally replacement of chondrocytes by osteoblasts and calcified tissue.^[^ ^77^, ^78^, ^79^ ^]^ While it is not possible to distinguish if the modest increase in DNA during coculture is due to hAC‐ or hMSC‐specific proliferation/apoptosis, or a combinations thereof, it indicates that the majority of cells remain in a healthy, low‐proliferative, chondrogenic differentiation phase throughout the culture period across all the investigated culture arrangements.

Interestingly, it was observed that the addition of hMSCs to hACs in the direct coculture (mixed) condition displayed a collagen turnover of the newly synthesized matrix from collagen I to collagen II over the culture period (Figure S2, Supporting Information). This is consistent with the early matrix production seen with hACs followed by the late contribution from hMSCs. This provides evidence to support previous suggestions that in hAC and hMSC cocultures, hMSCs can protect chondrocytes from apoptosis as well as loss of chondrogenic phenotype via paracrine effects and release of growth factors, such as TGF‐*β*3, IGF‐1, and FGF‐2.^[^ ^32^, ^80^ ^]^ The intensified Cx43‐mediated communication noted in the hMSC containing cultures may thus be responsible for the phenotypic modulation observed.^[^ ^58^, ^81^, ^82^ ^]^ An overexpression of Cx43 has specifically been suggested to reprogram chondrocytes into a more stem‐like state, by increasing vimentin, N‐cadherin, and the surface markers CD105 and CD166, activating a chondrocyte–mesenchymal transition.^[^ ^81^, ^82^, ^83^, ^84^, ^85^ ^]^ It was also demonstrated that hACs can help modulate the stability of hMSCs, reducing the risk of developing hypertrophic cartilage, which has been suggested to be due to the secretion of parathyroid hormone related proteins.^[^ ^35^, ^81^ ^]^

The importance of reduced hypertrophy, upregulated paracrine signaling, coordinated cell migration, and rapid fusion through hMSC and hAC cocultures was highlighted by 1) secretion of collagen type X within MSC monocultures, and 2) outgrowth of fibrocartilage tissue without cell nuclei otherwise observed in the pure hAC cultures. Considering the above aspects, the performance of pure hAC spheroids raises several concerns for long‐term functionality and translational capacity of autologous hAC spheroids. The reason for this is that fibrocartilage without chondrocytes is considered as a trademark of degenerative cartilage diseases.^[^ ^86^, ^87^ ^]^ This study further underlines that utilizing MSCs risks hypertrophic development and subsequently endochondral ossification, also when cocultured indirectly with hACs. With hAC tissues mimicking dense native cartilage tissue within this indirect coculture model, the reduced ability of potent hMSCs to fuse with hAC spheroids further revealed that MSCs are not able to drive functional fusion with mature cartilage tissues fully on their own. This limited fusion observed in both the hAC–hAC and hAC–MSC conditions reflects the major clinical challenge that is integration, especially in a lateral direction. ^[^ ^8^, ^9^, ^88^ ^]^ Together, this data offers the first proof‐of‐concept for an in vitro 3D‐model to reliably study lateral fusion mechanisms between multicellular spheroids as well as engineered and mature cartilage tissues.

When studying the samples from an extended macroscale perspective, investigating the constructs as a whole and not just limited to the tissue fusion region, immunohistochemistry and gene analysis revealed no significant difference between any of the four conditions in terms of collagen type I and aggrecan expression. It is thus clear that very different responses can be observed depending on local microenvironments. Cells within the prematured tissue spheroids are densely packed while cells within the fusion region are relatively sparse and likely had greater access to both oxygen and nutrients. RNA results from the whole constructs again suggest that direct (mixed) coculture of MSCs and hACs is a more potent strategy, compared to indirect cocultures (hAC/hMSC), to activate collagen type II synthesis to a similar level to that of pure hMSC cultures. Of a particular note, higher SOX9 expression was observed in pure hAC cultures compared to both pure hMSC and hAC/hMSC (indirect coculture) conditions. Although SOX9 is known to be an essential component of the cartilage differentiation pathway, it is also known to be dynamically expressed.^[^ ^89^ ^]^ SOX9 is specifically required for collagen type II expression, which has already been upregulated in hMSC spheroids, and could thus explain the subsequent lower SOX9 expression after 4 weeks of continued culture. Due to this dynamic and biphasic gene expression, previous reports suggest that a decrease in SOX9 can in some cases indicate a more matured cartilaginous tissue.^[^ ^90^, ^91^ ^]^ It should also be noted that high levels of SOX9 expression can lead to inhibition of the collagen type II gene,^[^ ^92^ ^]^ which was observed in the hAC pure cultures. While the 3D‐models offer the flexibility in analyzing both the constructs as a whole or spheroids as individual zones, it should be noted that it is of importance to study the interface region separately as the biological fusion developments may be masked when analyzed on a macroscale or organ level.

From a tissue fusion perspective, this study collectively suggests that, while hACs are able to proliferate and secrete new fibrocartilage in the fusion region, the addition of hMSCs is required to help modulate cellular phenotype and ensure that the fusion region is filled with hyaline cartilage tissue as well as migratory cells. The power of using coculture systems, besides modulating the quality of new repair tissue, is that it may also overcome cell source limitations and costly two‐stage surgery processes with traditional autologous chondrocyte implantation‐based strategies. By providing a bridge between understanding cellular differentiation and tissue fusion, organ growth as well as host integration, the high‐throughput 3D‐bioassembly model developed in this study may be able to help in the quest to design therapies with more advantageous outcomes for patients with cartilage defects. This hybrid 3D‐bioassembly approach, combining spheroids and thermoplastic 3D scaffolds, can further be thought of as a modular approach to design clinically relevant replacement grafts. The scaffold herein provides a “structural module” providing the mechanical properties responsible for shock absorption, load bearing, and appropriate load transmission as well as implant fixation possibilities and enabling handling and defining and preserving overall construct shape. In addition, the scaffold fabrication technique enables designed pores for placement of the tissue modules. The spheroid subsequently provides cellular modules responsible for cell seeding, distribution and ECM production, fusion, integration with surrounding native tissue, and eventual maturation of the construct. Ultimately, assembling cartilage spheroids within 3D‐plotted scaffolds further allows for control of the tissue unit locations, enabling the biofabrication of complex tissues on a clinical scale.^[^ ^46^ ^]^

As a proof‐of‐concept, this tailorability of the modular tissue units was investigated through the utilization of an extensive library of biomaterials available today enabling precise control over the cellular microenvironment; ranging from oxygen, nutrients, and stiffness gradients to the delivery of bioactive factors.^[^ ^47^, ^50^, ^51^, ^52^, ^93^, ^94^, ^95^, ^96^, ^97^, ^98^, ^99^, ^100^, ^101^, ^102^, ^103^ ^]^ This platform subsequently allowed the study and optimization of the factors that influence the fusion of engineered tissue implants with the surrounding host tissue, including enzymatic or hydrolytic biomaterial degradation, biomaterial stiffness, biomaterial composition as well as cell type and density. The use of three distinct cell types further highlights that the developed fusion model can be applied to study developmental biology processes of various joint tissues. Taken together, the developed 3D‐model provides a versatile and high‐throughput platform to help bridge the knowledge gap between engineering tissues and the potential fabrication of organ‐like structures.

## 5 Conclusions

In this study, melt extrusion of PCL was successfully fabricated into 3D scaffolds enabling designed pores for precise placement of modular tissue spheroids. Multicellular cartilage spheroids were furthermore successfully fabricated using a 96‐well format and 3D‐bioassembled into hybrid constructs. We systematically studied the macro‐ and microscale fusion‐kinetics and ‐mechanism of multicellular spheroids in these 3D‐bioassembled hybrid tissue constructs following direct or indirect coculture. Overall, coordinated migration was observed to be an essential part of hMSC tissue fusion mechanisms while the newly formed tissue interface of hACs was lacking both cell nuclei and collagen type II. The addition of hMSCs to hACs, both as direct and indirect cocultures, was observed to increase phenotypic stability in the fusion region, possibly through increased paracrine signaling. Close contact between hMSCs and hACs (mixed) was furthermore observed to be superior over more distant, indirect cultures (hACs/hMSCs) in terms of upregulating GAG/DNA compared to pure hAC cultures while maintaining collagen II production similar to pure hMSC cultures and minimizing collagen type X deposition. This study thus demonstrated that combining high‐throughput fabrication of cartilage tissue spheroids with extrusion‐based 3D printing of thermoplastic polymer scaffolds offers an adaptable route to study 1) multicellular cell–cell interactions and cell differentiation capacity in both a micro‐ and macroscale, 2) multicellular tissue fusion mechanisms in developmental stages of tissue growth, and 3) fusion strategies for 3D‐bioassembly technologies and integrative repair strategies. As such, this hybrid 3D‐bioassembly approach offers an attractive in vitro 3D‐model to probe fundamental questions in developmental biology as well as addressing major clinical challenges that is cartilage tissue growth, fusion, and implant integration. The flexibility of the platform furthermore allows the study of various joint tissue types as well as biomaterial‐based spheroids for controlled growth factor or drug delivery. It may thus provide a steppingstone toward unlocking the full potential of anisotropic tissues and organ‐scale biofabricated constructs and regenerative medicine by facilitating a better understanding of the underlying mechanisms behind developmental biology, cellular crosstalk, and tissue fusion of various joint tissues for example.

## Conflict of Interest

The authors declare no conflict of interest.

## Supporting information

Supporting Information

## Data Availability

The data that support the findings of this study are available from the corresponding author upon reasonable request.
