# Supplementary material for: Probing Multicellular Tissue Fusion of Cocultured Spheroids—A 3D‐Bioassembly Model
Source: Adv Sci (Weinh). 2021 Oct 10;8(22):2103320. doi: 10.1002/advs.202103320 (PMC8596109; doi:10.1002/advs.202103320)
Supplement: Supplementary file 1 — Supporting Information [file ADVS-8-2103320-s001.pdf]

WILEY-VCH

© 2021 Wiley-VCH GmbH

ADVANCED SCIENCE
Open Access

Supporting Information

for *Adv. Sci.*, DOI: 10.1002/advs.202103320

Probing Multicellular Tissue Fusion of Co-Cultured Spheroids - a 3D Bioassembly Model

Gabriella C.J. Lindberg, Xiaolin Cui, Mitchell Durham, Laura Doornkamp-Veenendaal, Ben Schon, Gary J. Hooper, Khoon S. Lim, and Tim B.F. Woodfield**

Supporting Information

Probing Multicellular Tissue Fusion of Co-Cultured Spheroids - a 3D Bioassembly Model

Gabriella C.J. Lindberg, Xiaolin Cui, Mitchell Durham, Laura Doornkamp-Veenendaal, Ben Schon, Gary J. Hooper, Khoon S. Lim, and Tim B.F. Woodfield**

1. Cell migration

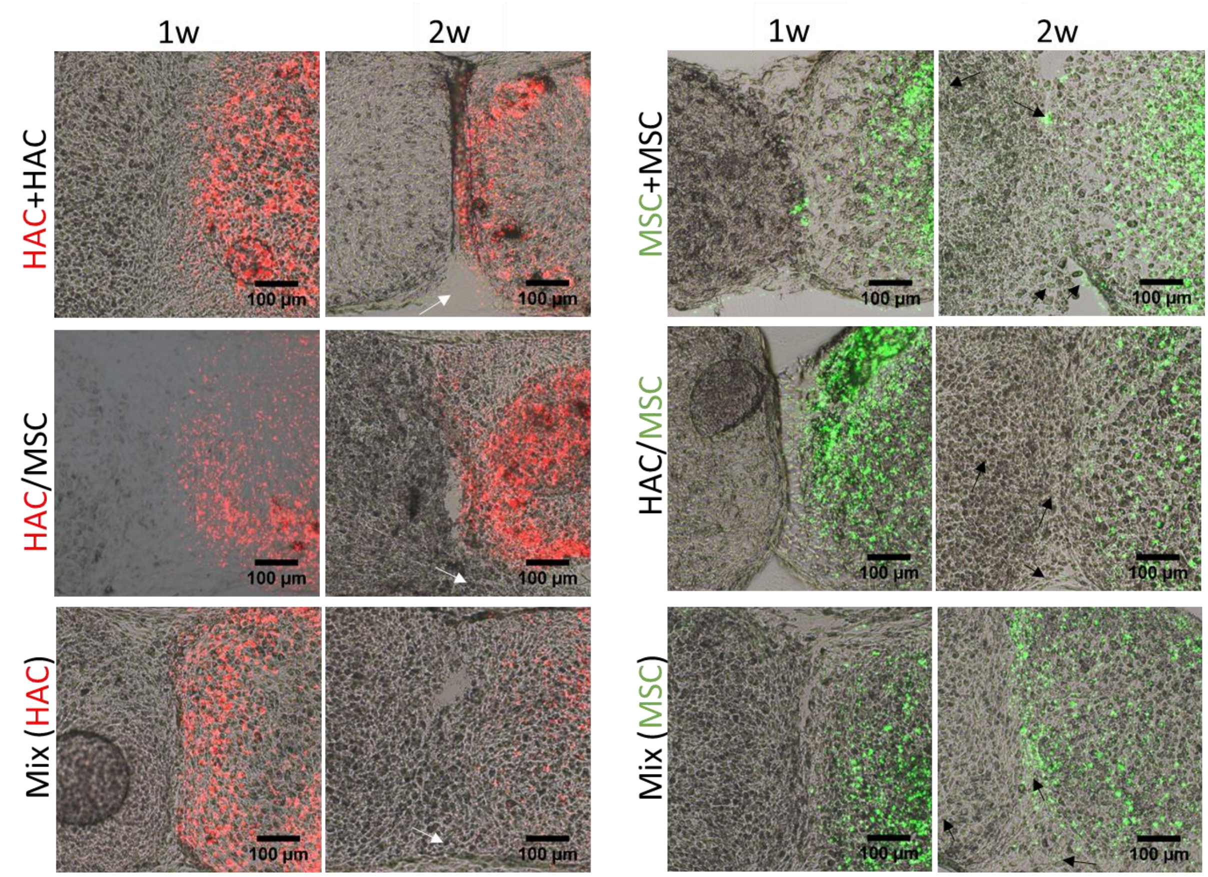

Figure S1. Histological sections through the centre of samples with fluorescently labelled cells overlaid onto brightfield images as a function of time. HACs (red) were not detected in the fusion zones while hMSCs (green) displayed a high migratory capacity and were found present in most parts of the newly secreted matrix.

2. Matrix Formation

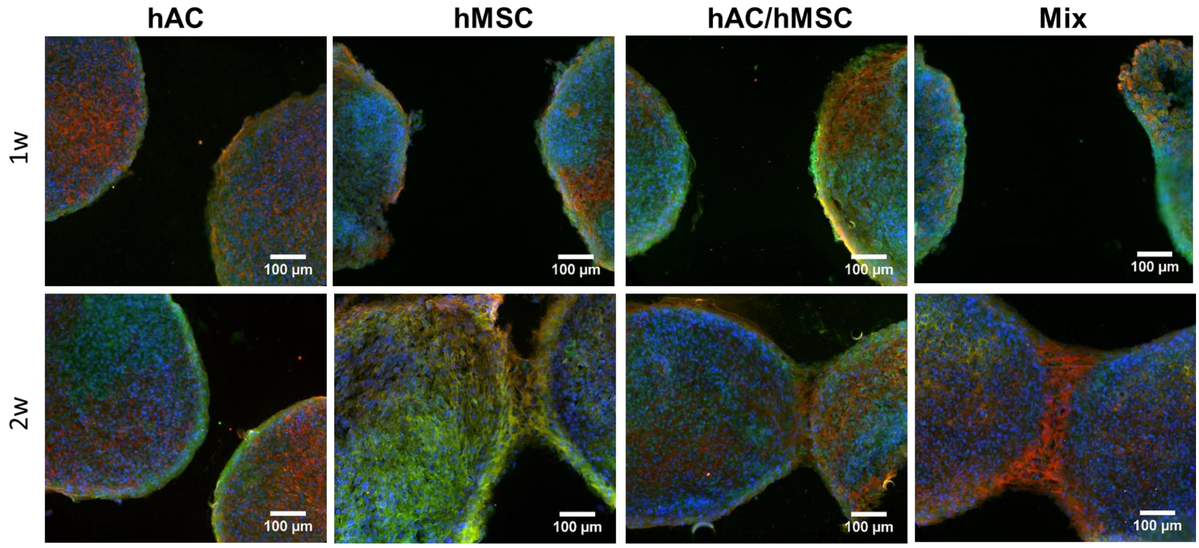

Figure S2. Immunohistological sections through the centre of samples stained with Collagen type II (green), Collagen type I (red) and DAPI (blue), as a function of time.

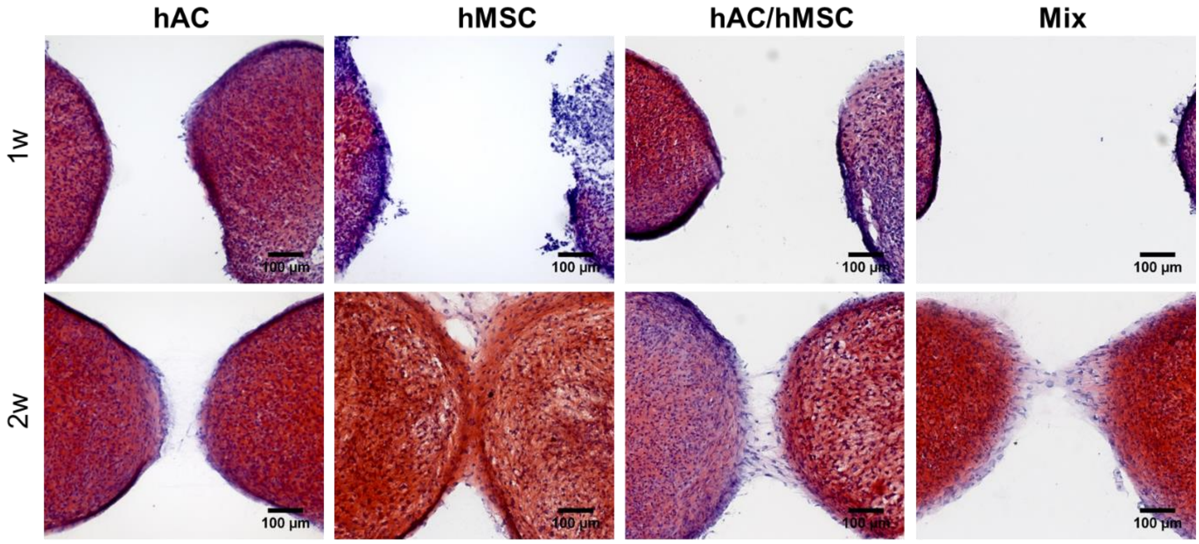

Figure S3. Histological sections through the centre of samples stained with safranin-O/fast green, as a function of time.

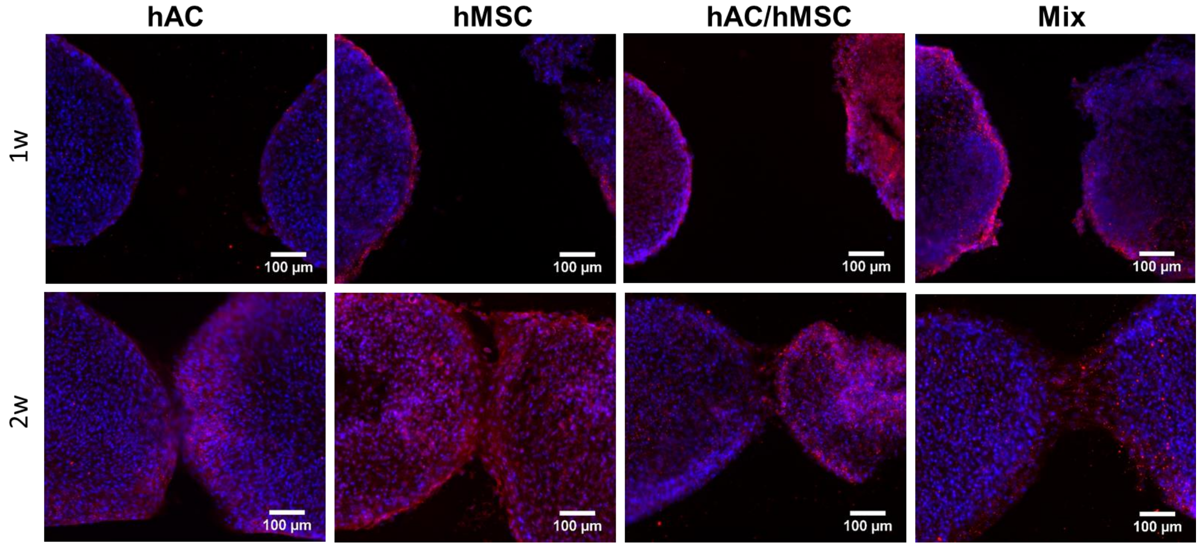

Figure S4. Immunohistological sections through the centre of samples stained with Connexin 43 (red) and DAPI (blue), as a function of time.

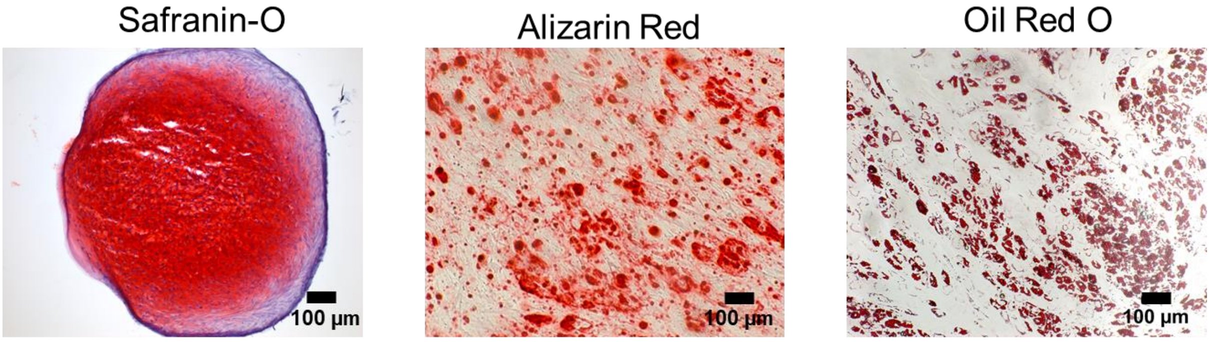

Figure S5. Multi-lineage potential of MSCs was confirmed by a tri-lineage differentiation (adipogenic, osteogenic, and chondrogenic) assay. After 3 weeks in vitro, samples were stained for adipogenic (Oil Red O), osteogenic (Alizarin Red), and chondrogenic (Safranin O) differentiation markers.

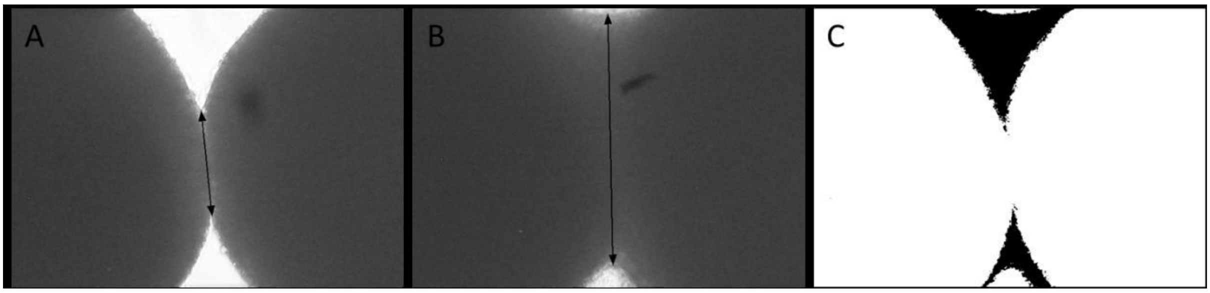

Figure S6. Light microscopy image of scaffold with spheroids. Measurement of fusion front and area of change from image A (t = 7 days of accumulative culture) to image B, is shown in C.

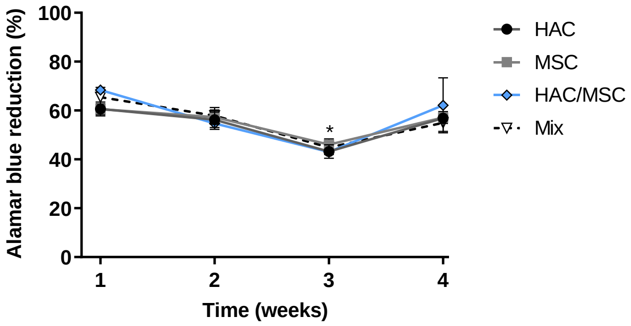

Figure S7. AlamarBlue® was used to quantify the metabolic activity of bioassembled spheroids. Briefly, each well was incubated with 330μl AlamarBlue solution for 1h 20min. The reduction of AlamarBlue® in the solution was determined by reading absorbance at wavelengths of 570 nm and 600 nm using a spectrophotometer (Thermo Scientific Varioskan Flash) followed by data processing according to manufacturer's instructions. A slight reduction in metabolic activity was observed at 3 weeks of culture for all three conditions. The decreased metabolic cell activity was however not related to any decrease in matrix formation or DNA. This may simply reflect a lowered permeability of the resazurin dye into the tissue as new matrix is being formed over time. In addition, cells residing in mature cartilage are well-known to have low metabolic activity due to low mitochondrial numbers, and are subsequently able to synthesize ECM components in low oxygen environments with low metabolic turnover. The observed change in metabolic activity may thus be due to any number of events in the healthy and sequential biological processes to regenerate musculoskeletal tissue ranging from growth, maintenance, maturation and repair.
